# Plant Stress Detection Using a Three-Dimensional Analysis from a Single RGB Image †

**DOI:** 10.3390/s24237860

**Published:** 2024-12-09

**Authors:** Madaín Pérez-Patricio, J. A. de Jesús Osuna-Coutiño, German Ríos-Toledo, Abiel Aguilar-González, J. L. Camas-Anzueto, N. A. Morales-Navarro, J. Renán Velázquez-González, Luis Ángel Cundapí-López

**Affiliations:** 1 Department of Science, Tecnológico Nacional de México/IT Tuxtla Gutiérrez, Carr. Panamericana 1080, Tuxtla Gutierrez 29050, Chiapas, Mexico; mperez@ittg.edu.mx (M.P.-P.); german.rt@tuxtla.tecnm.mx (G.R.-T.); jcamas@ittg.edu.mx (J.L.C.-A.); nestor.mn@tuxtla.tecnm.mx (N.A.M.-N.); josue.velazquez@ittuxtlagutierrez.edu.mx (J.R.V.-G.); m13270146@tuxtla.tecnm.mx (L.Á.C.-L.); 2 Department of Computer Science, Instituto Nacional de Astrofísica, Óptica y Electrónica, Luis Enrique Erro 1, Cholula 72840, Puebla, Mexico; abiel@inaoep.mx

**Keywords:** plant stress detection, plant stress phenotyping, deep learning, visual pattern

## Abstract

Plant stress detection involves the process of Identification, Classification, Quantification, and Prediction (ICQP) in crop stress. Numerous approaches exist for plant stress identification; however, a majority rely on expert personnel or invasive techniques. While expert employees demonstrate proficiency across various plants, this approach demands a substantial workforce to ensure the quality of crops. Conversely, invasive techniques entail leaf dismemberment. To overcome these challenges, an alternative is to employ image processing to interpret areas where plant geometry is observable, eliminating the dependency on skilled labor or the need for crop dismemberment. However, this alternative introduces the challenge of accurately interpreting ambiguous image features. Motivated by the latter, we propose a methodology for plant stress detection using 3D reconstruction and deep learning from a single RGB image. For that, our methodology has three steps. First, the plant recognition step provides the segmentation, location, and delimitation of the crop. Second, we propose a leaf detection analysis to classify and locate the boundaries between the different leaves. Finally, we use a Deep Neural Network (DNN) and the 3D reconstruction for plant stress detection. Experimental results are encouraging, showing that our approach has high performance under real-world scenarios. Also, the proposed methodology has 22.86% higher precision, 24.05% higher recall, and 23.45% higher F1-score than the 2D classification method.

## 1. Introduction

As the world’s population continues to grow, the demand for food is increasing daily. To maximize crop yields, farmers must ensure that crops are grown under optimal conditions concerning temperature and nutrition. An alternative for increased production is the use of plant stress detection. This technology allows the identification, classification, quantification, and prediction of stress. Also, early identification of plant stress can help farmers to identify and address plant health issues before they become severe. This can prevent crop loss and improve the quality and quantity of the harvest [1]. Due to these characteristics, crop stress detection has been widely applied in various fields, including agriculture [2], forestry [3], horticulture [4], and ecology [5].

There are several approaches to plant stress identification. In most cases, these techniques use qualified experts who identify stress through visual (NON-VISUAL) symptoms [6,7,8]. In general, expert employees have good performance in different plants. Furthermore, this technique does not require specialized equipment, and it can detect changes in plants in real time. However, the disadvantages include subjectivity, i.e., the interpretation of visual characteristics may vary among observers [9,10]. Also, this alternative requires sufficient staff to guarantee quality crops.

Invasive techniques for plant stress detection involve the extraction of plant tissue samples for subsequent analysis. The most common techniques include the analysis of chlorophyll, proteins, carbohydrates, etc. [11,12]. These techniques provide detailed and accurate information about the plant’s status, which can be useful for decision-making in crop management. However, they can limit the ability of farmers and researchers to perform stress tests on plants on a large scale or in the field [13,14,15]. Moreover, the potential damage to the plant may affect its ability to grow and produce, which may have a negative impact on crop production.

The use of acoustic emission is another approach to detecting stress in crops [16,17]. This approach involves using acoustic emission sensors that measure root growth within the soil. These sensors detect changes in the speed and direction of root growth. Furthermore, this technique is non-invasive, i.e., this is not necessary to extract plant samples. However, acoustic measurements can be affected by water disturbance both internal and external to the plant, leading to inaccurate measurements.

Computer vision has significant progress in plant stress detection. Vision is a non-contact and non-destructive technology for image processing [18,19] and hyperspectral analysis (non-invasive) [20,21]. This technique uses devices that provide physical characteristics of the plant using image sensors such as digital, fluorescence, thermography, multispectral, etc. These approaches are commonly used because they do not destroy the sample studied and do not require direct contact. Moreover, this technique can analyze large crop areas in a short time, making it suitable for large-scale applications. However, the accuracy of the results may be affected by factors such as weather conditions, camera angle, and lighting.

Finally, there is significant progress in crop stress recognition using machine learning [22,23,24,25,26]. This was achieved via learning algorithms that learn the relationship between visual appearance and plant stress diagnosis. Unlike the other trends, this approach analyzes the plant without qualified labor dependency or crop dismemberment but adds the challenge of interpreting image ambiguities. Motivated by the results of learning algorithms and the potential benefits that image analysis provides, this work focuses on plant stress recognition using deep learning and visual patterns of plant geometry. In particular, we are interested in the 3D information in crop environments. We use a 3D methodology since a 2D approach can only recognize stress in images where the plant shows obvious physical signs of stress. However, in a plant with the first day of stress, the physical signs are not visually visible, whereas the decline of the leaves is one of the first morphological symptoms of crop stress. This information allowed our 3D methodology to recognize stress even when visual signals had not appeared on the plant. Also, the proposed method allows plant stress detection using visual characteristics of a single RGB image. We can deploy the methodology across multiple devices using an RGB camera, improving portability and reducing implementation costs. These devices enable remote implementation of the method, facilitating its adoption in rural areas. To our knowledge, the proposed approach is the first work to detect stress in crops using 3D information from a single RGB image.

## 2. Related Work

In this section, we present an overview of the current state of the art regarding the detection of plant stress using computer vision methods. Typically, crop stress is detected using visual characteristics and machine learning algorithms, including color, texture, and geometric shape, among others. In contrast, there has been a significant advancement in stress detection using deep learning techniques. Therefore, we have included two subsections to discuss stress estimation: one focusing on machine learning algorithms and the other on deep learning.

### 2.1. Algorithms That Use Machine Learning Algorithms

Color is one of the most commonly used features in machine-learning algorithms for plant stress detection [27,28,29]. This is because leaves usually have color changes due to sun exposure, soil pH, nutrient deficiency, or excess. For example, nitrogen deficiency in plants can cause green leaves to turn yellow due to premature wilting. Furthermore, color features are relatively easy to extract with efficient processing. However, these techniques are sensitive to changes in lighting and reflections, which produces coloration variations between similar images. This limitation is particularly significant in outdoor planting environments where lighting conditions are variable.

Using texture features in machine learning algorithms can help detect anomalies in the morphology of plant leaves [30]. For example, texture features can provide information about the roughness, granularity, and coarseness of the leaf surface, which can be useful for identifying deformations or pathologies that alter plant stress. On the other hand, these features are less sensitive to changes in lighting and reflections, which provides robustness in variable lighting conditions. However, texture features can be sensitive to scale and rotation. For instance, if an image of a leaf is taken from different angles, the texture pattern can vary dramatically, making it difficult to detect anomalies in the leaf. Similarly, when an image is zoomed out, texture patterns can become distorted due to blurring.

The use of geometric shape features (form-based features) can provide information about plant stress [31,32]. The shape of the leaf can vary in response to stress factors such as water deficiency, nutrient deficiency, and exposure to extreme temperatures. For example, under stress conditions, leaves may curve, curl, or have irregular edges. In most cases, these works use algorithms such as Canny, Hough transform, the Laplacian of Gaussian, etc. However, generalizing a specific geometric shape to a stress factor is complex, since leaves can vary in shape and size, providing infinite combinations. The lack of generalization of geometric shapes can negatively affect the learning algorithms. The latter is because generalization is essential to detect patterns in new images. Although these learning approaches with visual features provide remarkable advancement. These approaches are limited to extracting particular features. This limitation is particularly significant since using a single feature increases their sensitivity to environmental conditions or their difficulty in generalization.

We have previous work on plant stress using visual characteristics and machine learning [33]. In this work, we proposed a new approach for estimating chlorophyll contents in a plant leaf using reflectance and transmittance as base parameters. First, we proposed a novel optical arrangement to extract the base parameters. Also, we estimate the chlorophyll content using a learning algorithm where the inputs are the reflectance and transmittance. This approach provides significant advances in the processing of reflectance information and the transmittance of the sheet. However, in this proposal, it is necessary to dismember the leaves for the analysis of the crop.

### 2.2. Estimation of Stress Using Convolutional Neural Networks

Deep learning algorithms generate high-precision models for plant stress detection and also allow the identification of other deficiencies [34,35,36,37]. These deficiencies may include nutrient deficiencies, water stress, diseases, etc. Additionally, these algorithms possess the capability to generalize from a training dataset and apply that knowledge to new plant images, enabling better adaptation to different conditions and species. However, the majority of these studies focus on processing RGB images. This limitation implies that these algorithms can only recognize stress in crops where the plant exhibits obvious physical signs of stress, such as changes in leaf color or shape. This can be a drawback as certain types of stress can be more subtle and may not necessarily manifest as visual characteristics. Therefore, further exploration and development of techniques are required to enable stress detection in crops beyond superficial visual features.

In current works, there is significant progress in plant detection using depth sensors and learning algorithms [38,39]. These works focus on the three-dimensional reconstruction of the plant’s fruits. These approaches leverage the capability of depth sensors to capture detailed three-dimensional information about the shape and structure of the fruits. However, these works primarily focus on applications related to crop grasping and manipulation, overlooking stress analysis. These approaches are more oriented towards optimizing crop harvesting and assessing fruit ripeness. As a result, there is a research gap in fully harnessing the potential of three-dimensional information and deep learning for stress analysis and detection in plants. It is necessary to develop more comprehensive approaches that combine three-dimensional reconstruction with the visual features of the crop. The stress analysis will provide a complete assessment of the health status of plants. In addition, it will have significant applications in the optimization of cultivation methods and agricultural decision-making.

We have previous work on 3D volumetric reconstruction from a single RGB image [40,41,42]. While this methodology enables the creation of a 3D model, our primary focus has been on the volumetric extraction of urban structures, such as buildings, floors, and houses. In contrast, when it comes to the 3D extraction of strawberry leaves, the volume information is nearly nonexistent. In other words, while this information can be inferred, it does not significantly contribute to the performance of crop representation. Furthermore, the CNN network employed solely recognizes objects comprising urban structures, removing green areas in the analysis.

We also have prior research in the field of plant analysis using 3D information and deep learning [2]. In this work, we use a depth sensor for 3D reconstruction of the crop. While the use of depth sensors simplifies 3D extraction, it also restricts their applicability to indoor environments and escalates implementation costs. For example, these sensors are prone to fail under outdoor scenarios due to the sun’s radiation. Also, they are not integrated into personal devices (such as mobile phones, personal assistants, or personal computers). Lastly, their power consumption (in watts), cost, and size are higher than those of RGB sensors.

In this work, in contrast to the related work, the proposed methodology conducts plant stress inference and their 3D reconstruction using a sole RGB image. By employing an RGB camera for crop analysis, we can deploy the methodology across multiple devices, improving portability and reducing implementation costs. Furthermore, our results surpass the performance of previous research involving 3D or 2D information. Finally, we use a 3D methodology since a 2D approach only can recognize stress in images where the plant shows obvious physical signs of stress. However, in a plant with the first day of stress, the physical signs are not visible visually, whereas the decline of the leaves is one of the first morphological symptoms of crop stress. This information allowed our 3D methodology to recognize stress even when visual signals have not appeared on the plant.

## 3. The Proposed Methodology

**Figure 1:**
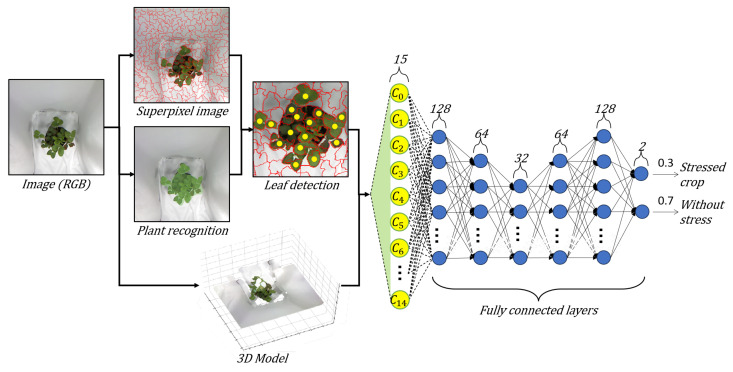
Block diagram of the proposed methodology.

We propose a methodology for plant stress detection using 3D reconstruction and deep learning from a single RGB image. Our approach is to predict plant stress by leveraging its three-dimensional geometry, which we achieve by combining the abstraction power of deep learning with information about the plant’s height and 3D structure. This work brings the best of the two worlds to address the problem of plant stress. The methodology consists of three steps. First, we use plant recognition to segment, localize, and delimit the crop (Section 3.1). Second, we apply leaf detection analysis to classify and find the boundaries between leaves (Section 3.2). Finally, we use a Deep Neural Network (DNN) and the 3D reconstruction for plant stress detection (Section 3.3). Figure 1 shows the schematic representation of the proposal.

### 3.1. Plant Recognition

**Figure 2:**
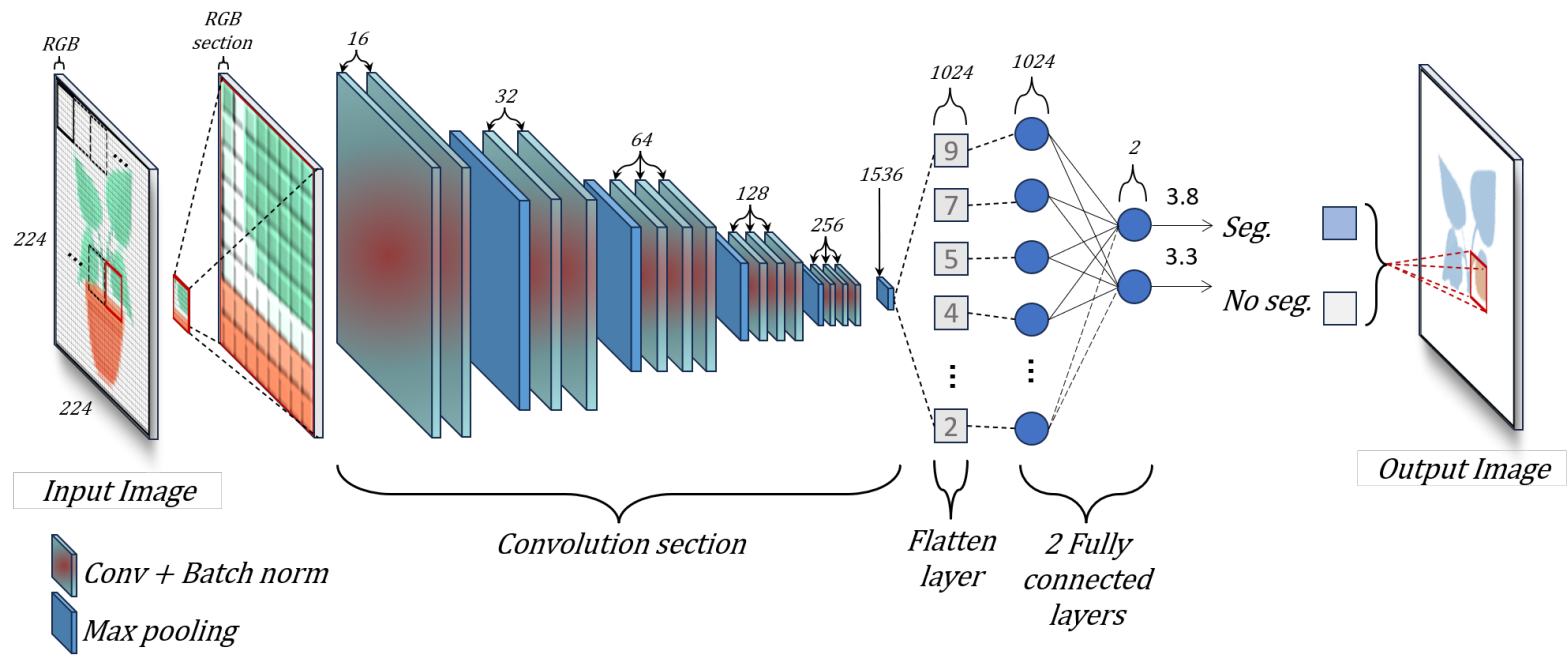
Architecture of our CNN for semantic segmentation.

Our plant recognition step provides the segmentation, location, and delimitation of the crop in the image. For that, we propose a Convolutional Neural Network (CNN) configuration, where the input is an RGB section and the output is a segmented pixel. Using this configuration, we can convert an object detection network with bounding box into a semantic segmentation network. On the other hand, our semantic segmentation architecture allows us to convert a dataset with few images into a big training set. Figure 2 shows an example of our CNN configuration.

#### 3.1.1. Training Set

In the training set of semantic segmentation, we use the “Eschikon Plant Stress Phenotyping Dataset” [43] and a proposed dataset of strawberry plants (Section 4). In these datasets, we develop the ground truth of semantic segmentation, i.e., we perform manual semantic labeling of the images. For that, we use two labels (plant $\upsilon^{1}$ and no-plant $\upsilon^{2}$). To obtain the training set, we divide the images of the datasets with labels (plant $\upsilon^{1}$ and no-plant $\upsilon^{2}$) in RGB images of 32 × 32 pixels. For example, we can obtain 630 small sections (32 × 32 pixels) using an image (1920 × 1080 pixels). Using these small sections, we can convert a dataset with few images into a big training set.

#### 3.1.2. CNN for Semantic Segmentation

The input of the CNN is an RGB section Φ with a size of 32 × 32 pixels. Our network contains two stages. The first stage of the network (encoder) is based on the VGG16 architecture [44]. We use this encoder since it has promising results to obtain the feature map for the object classification tasks [45,46]. The first stage consists of 13 convolutional, 13 batch normalization, and 5 max pooling layers, which extract feature maps with different resolutions from the input image. This stage is the backbone of the network since the extracted features are shared in the second stage. The second stage combines all the found local features of the previous convolutional layers. We use a flatten layer, dense layers, and batch normalization. Finally, we use a second dense layer to obtain a label of the two possible labels (crop $\upsilon^{1}$ and no-crop $\upsilon^{2}$). Figure 2 shows the architecture of our CNN for semantic segmentation.

Our convolutional layers consist of several feature maps. Each feature map connects to the preceding layer via a kernel, i.e., a size-fixed weight matrix. In each iteration, this kernel performs a convolution operation on a group of neighboring neurons within a local area of the preceding layer. Then, the kernel slides with a fixed stride until this operation is performed on all neurons. After adding a bias to the convolution item, the output of the convolutional layer is activated by a nonlinear activation function like Rectified Linear Unit (ReLU), sigmoid and tanh, etc. Because of the ability to avoid vanishing gradient and the fast convergence speed, the ReLU function is chosen to be the activation function in our convolutional layer:

(1) $$y_{i}=ReLU\left(y_{i-1}*W_{i,i-1}+b_{i}\right)$$

(2) $$ReLU\left(x\right)=\left\{\begin{array}{c} x,\;x>0,\\ 0,\;x\leq 0 \end{array}\right.$$

where $y_{i-1}$ and $y_{i}$ denote the output of two successive convolutional layers respectively, $W_{i,i-1}$ denotes the connection weight matrix between them, * represents the operation of convolution, and $b_{i}$ refers to the bias. Reducing the resolution of the convolutional layer can preserve scale-steady features. Hence, the max pooling layer is introduced to carry out a down-sample operation after the convolutional layer. In the pooling layer, the down-sample operation aims to derive a unique statistic from the local region of the convolutional layer by taking the pooling strategy.

The prediction error between the output and the desired output is called the loss function. In the loss function, we use gradient descent, a popular optimization algorithm for neural networks. We employ Adaptive Moment Estimation (Adam) among the various gradient descent algorithm variants because it has fast convergence and is generally considered to be the best overall [47]. Using Adam to minimize the loss function, we can effectively train our neural network and improve its performance.

**Figure 3:**
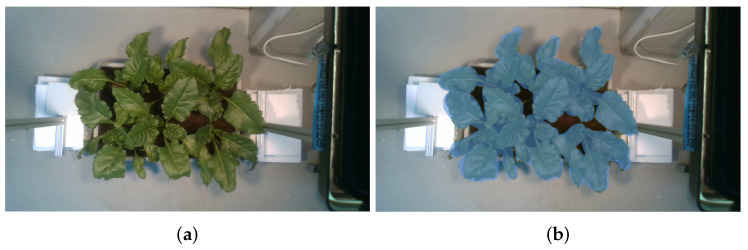
(**a**) RGB image. (**b**) Semantic segmentation. Our methodology paints pixels (light-green) that correspond to the plant foliage.

Finally, we propose a network configuration. For that, our architecture divides the input image into sections of 32 × 32 pixels, and the output is a semantic label. We use a sliding window with a sweep of one pixel. For example, if we consider an image of 116 × 116 pixels, our approach needs 100 processes to segment. Using this configuration, we can convert an object detection network with a bounding box to a semantic segmentation network. On the other hand, we have two semantic labels (plant $\upsilon^{1}$ and no-plant $\upsilon^{2}$). The CNN paints the central pixel ρ with green color if it has a plant label and with black color if it has a no-plant label. Figure 3 shows a qualitative result of our methodology in semantic segmentation.

We use VGG16 because its architecture is well across different domains, making it adaptable to various image recognition problems outside the specific domain of its original training [48]. The depth enables it to capture both low-level features (edges, textures) and high-level features (objects, shapes) effectively, making it versatile for various image recognition tasks. Also, this network has been used in different 3D reconstruction methodologies [2,40,41,42].

### 3.2. Leaf Detection

We propose a leaf detection analysis using superpixels to classify and locate the boundaries between the different leaves. This analysis obtains the superpixels to analyze. For that, this step has two components. First, we present the superpixel technique implemented. Second, we use our semantic segmentation and the superpixels to classify the sections with leaves.

#### 3.2.1. Superpixel Image

Our superpixel approach is Simple Linear Iterative Clustering (SLIC) [49]. For that, we denote the superpixel image as $I_{s}$, where $\psi_{i}$ denotes the $i^{th}$ superpixel and $\beta_{i}$ denotes the $i^{th}$ th superpixel in an image $I_{s}$. For the superpixel approach, we use the following parameters: desired number of superpixels = 999, number of pixel-level iterations = 9, and shape smoothing term = 5. These parameters are universal for all the images processed. The value of the desired number of superpixels considers the object number in the image. In our case, we use a high number of superpixels since we divide the plant into multiple leaf sections. These sessions increase the performance of our detection.

Table 1 presents model performance metrics as precision, recall, F1-Score, and accuracy across varying numbers of superpixels (800, 900, 999, 1100, and 1200). The precision values start at 0.9056 for 800 superpixels, rise to 0.9392 for 1100, and decrease to 0.9286 for 1200. recall increases progressively from 0.9314 at 800 superpixels, reaching its highest value of 0.9661 at 999 before a fine reduction to 0.9548 at 1200. F1-Score follows a similar tendency, starting at 0.9183, peaking at 0.9500 at 999 superpixels, and reducing barely later. accuracy improves from 0.9341 at 800 superpixels, peaking at 0.9591 at 999, and slightly decreasing to 0.9523 at 1200. The model obtains its best performance at around 999 superpixels.

#### 3.2.2. Superpixel Segmentation

The superpixel segmentation step classifies the superpixels in plant and no-plant. For that, we analyze the number of pixels with plant labels ($\upsilon^{1}$), where *i* denotes the $i^{th}$ superpixel $\psi_{i}$ in an image $I_{s}$, $\varphi_{i}$ is the number of pixels in a superpixel $\psi_{i}$, and $\gamma_{i}$ is the number of pixels with plant labels ($\upsilon^{1}$). If $\vartheta_{i}=1$, we consider the superpixel $\psi_{i}$ as a sheet, i.e., we use superpixel $\psi_{i}$ in our three-dimensional analysis (Section 3.3). Otherwise, we discarded the superpixel $\psi_{i}$ of our analysis. Our threshold function (*S*) is defined as

(3) $$S\left(\vartheta_{i}\right)=\left\{\begin{array}{c} 1\;\;\;\;\;\mathrm{if}\;\;\;\;\;\;\;\;\;\;\;\;\;\;\varphi_{i}=\gamma_{i},\\ 0\;\;\;\;\;\;\;\;\;\;\;\;\;\;\;\;\;\;\;otherwise, \end{array}\right.$$

### 3.3. Three-Dimensional Model Analysis

The 3D model analysis predicts plant stress by leveraging its three-dimensional geometry, which we achieve by combining the abstraction power of deep learning with information about the plant’s height and 3D structure. This analysis has three phases. The first uses a CNN to obtain the depth image inference. Second, we recover the plant’s three-dimensional geometry using the pinhole camera model. Finally, we propose a plant stress analysis from the 3D reconstruction and a Deep Neural Network (DNN).

#### 3.3.1. Depth Image Inference

There is significant progress in depth estimation from learning algorithms. This estimation uses deep learning that learns the relationship between visual features and depth information. In most cases, these visual features focus on the depth estimate of urbanized environments, i.e., these networks maintain their performance in particular scenarios. Unfortunately, this estimation decreases its performance in unfamiliar scenarios such as plant habitat. Motivated by this limitation, we propose training a convolutional network focused on depth detection in plants. In this case, we focus on this network at depth for strawberry plants. For that, we use our database for training (Section 4). Our database has images of strawberry plants and their depth images. On the other hand, we use our network configuration to learn the relationship between visual features and depth information (Section 3.1.2). Using this configuration, we can convert a dataset with few images into a big training set.

The input of our depth network is an RGB section $\Phi^{i}$ with a size of 32 × 32 pixels. This RGB section was related to the depth image to obtain its average gray-scale value. The depth images have a value range of 0 to 255, where the depth tends to 0 when the distance increases. In our experiments, we use morphological (Section 4.1) and dry weight analysis (Section 4.2) to obtain the heights of the leaves with and without stress. These analyses indicate that our stressed leaves maintained a distance of 75 to 80 cm.

#### 3.3.2. Pinhole Camera Model

We use the basic pinhole model to extract the 3D model. This model considers the projection of a point P(X,Y,Z) in space to a point p(x,y) in the image plane. The relative size of an object in the image depends on the image plane distance *Z* and the focal length *f*. The focal length *f* is the distance between the camera center $C_{o}$ (camera lens center) and the image plane. The optical center or principal point $O_{o}$ is the origin of coordinates in the image plane, but in practice, it may not be. By similar triangles, one quickly computes that the point P(X,Y,Z) is mapped to the point p(fX/Z,fY/Z,f) on the image plane.

We use the pinhole model with the image plane information and the depth of the sensor to compute the 3D recovery in crops. In our extraction, we convert the information in meters. For that, we divided the scale factor *k* with the maximum value 255 (mono image) and multiplied by a depth *z* (Section 3.3.1). The scale factor *k* is the maximum depth of the Kinect sensor (Section 3.3.1). For example, considering a maximum depth *k* of 4 m and a depth estimation *z* of 128 in gray-scale, *Z* is approximately 2 m. Equations (4)–(6) compute the coordinates (X,Y,Z) of a point in the space.

(4) $$Z=\frac{k\times z}{255}$$

(5) $$X=\frac{x\times Z}{f}$$

(6) $$Y=\frac{y\times Z}{f}$$

#### 3.3.3. Plant Stress Phenotyping

Using the 3D plant model, we calculate the 3D centroid of the detected leaves (Section 3.2). This centroid provides a 3D compact representation of the leaf. For that, we use a simplification of the intensity centroid. This simplification of intensity centroid obtains the central point by 3D leaf extracted. We define the moments as

(7) $$m_{p,q,g}^{j}=\sum_{X,Y,Z}^{w}X^{p}Y^{q}Z^{g}$$

where *j* denotes the $j^{th}$ 3D leaf extracted and *w* is the number of pixel projections by leaf. On the other hand, (p,q,g) are the orders of the moment $m_{p,q,g}^{j}$ (we use an order of 0 or 1). We determined the centroid of the 3D leaf as

(8) $$C^{j}=\left(\frac{m_{1,0,0}^{j}}{m_{0,0,0}^{j}},\frac{m_{0,1,0}^{j}}{m_{0,0,0}^{j}},\frac{m_{0,0,1}^{j}}{m_{0,0,0}^{j}}\right)$$

#### 3.3.4. DNN for Plant Stress Detection

**Figure 4:**
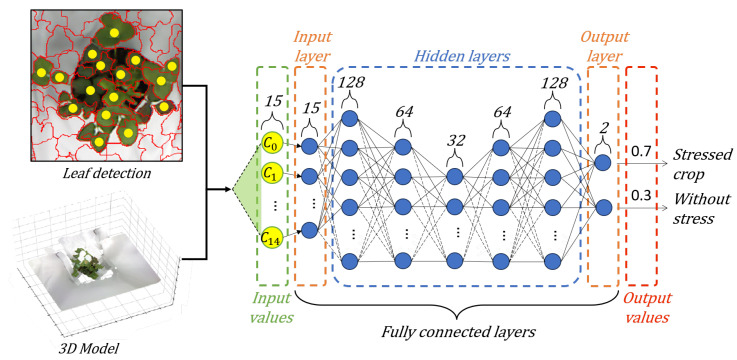
DNN for plant stress detection.

We performed an exhaustive exploration of DNN architectures to minimize the classification errors in the proposed methodology. We carried out different combinations of hidden layers and neurons. Through this process, an efficient and optimal DNN architecture emerged, characterized by one input layer of fifteen neurons, five hidden layers (128, 64, 32, 64, 128), and one output layer (2). Figure 4 shows our architecture for plant stress detection.

Our DNN has an input layer consisting of fifteen neurons. These neurons use the characteristic vector $C_{j}$, which corresponds to fifteen centroids of the 3D leaf. Then, the hidden layers adjust the weights between the neural connections to perform predictions to minimize the difference to the correct labels. This allows the DNN to learn and adapt to the complexity of the input data. The output layer consists of two neurons that classify the plant stress using a sigmoid activation function.

We use the sigmoid function in the output layer because it is suitable for binary classification problems—that is, this methodology classifies only two classes. The sigmoid function is represented by Equation (9), where $y_{i}$ and $y_{i-1}$ represent the outputs of the two connected layers. The sigmoid function transforms the values entered into a (0, 1) scale, where 1 indicates the stressed crop and 0 indicates without stress in Equation (10).

(9) $$f\left(y_{i}\right)=sigmoidal\left(\frac{1}{1-e^{-y_{i-1}}}\right)$$

(10) $$sigmoidal\left(x\right)=\left\{\begin{array}{c} 1,x>0\\ 0,x\leq 0 \end{array}\right.$$

## 4. Strawberry Plant Stress Phenotyping Dataset

Our dataset presents the different stress levels in strawberry plants. We began the development of our dataset when the plants showed morphological signs of stress. In our dataset, each RGB image has a depth image. This dataset has 1440 depth images and 1440 RGB images of 640 × 480 pixels captured during 15 days. Also, we use coconut substrate due to its properties in water retention, aeration, and pH. We develop eight sets of plant stress detection (A, B, C, D, E, F, G, H). For that, we classified the sets according to the type of irrigation: plants with water irrigation (A, B, C, D); plants with water irrigation and NPK (Nitrogen, Phosphorus, and Potassium) nutrients (E, F, G, H). In each irrigation dose, we use 250 mL of water or 250 mL of water and 17% NPK nutrients.

Group A has plants with excess water (irrigation daily). Group B is the plants with water deficit (irrigation with five-day intervals). Group C considers the plants without water stress (irrigation with two-day intervals). Group D is moderate water stress (irrigation with three-day intervals). On the other hand, group E has plants with excess water and nutrients (irrigation daily). Group F is the plants with water and nutrients deficit (irrigation with five-day intervals). Group G considers the plants with moderate water and nutrient stress (irrigation with two-day intervals). Group H is the plants without hydric stress (irrigation with three-day intervals). Table 2 shows the characteristics of each group. While we work with an open dataset (Eschikon Plant Stress Phenotyping Dataset [43]), a significant limitation is the absence of depth information regarding the plants. This aspect is vital in our methodology since we need to be able to analyze the three-dimensional inference, i.e., we need to compare our reconstruction with the 3D ground truth of the crop. Furthermore, considering this information can allow the use of 3D information in other applications such as crop manipulation, plant pruning, or fruit harvesting.

### 4.1. Plant Morphological Analysis

**Figure 5:**
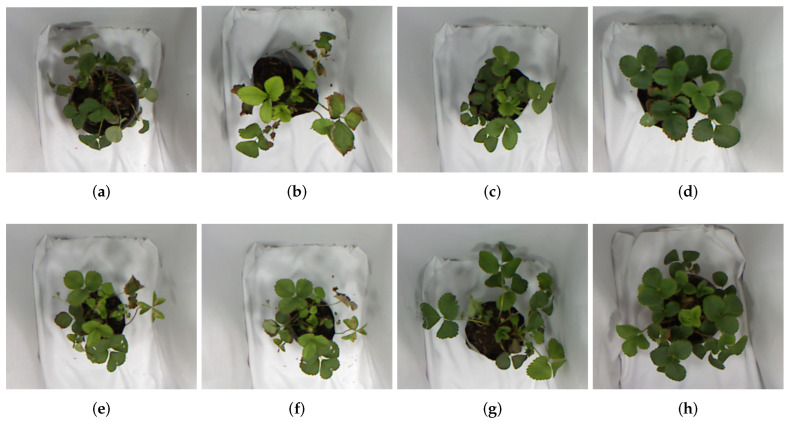
We developed eight sets of plant stress detection (A, B, C, D, E, F, G, H). For that, we classified the sets according to the type of irrigation: plants with water irrigation (A, B, C, D); plants with water irrigation and NPK (Nitrogen, Phosphorus, and Potassium) nutrients (E, F, G, H). The groups (A, B, E, F) have symptoms of severe damage to the leaves. Groups (D, G) showed stress symptoms. However, the leaves of sets (C, H) were firm and healthy due to adequate watering. (**a**) Excess water (Group A). (**b**) Water deficit (Group B). (**c**) Without water stress (Group C). (**d**) Moderate water stress (Group D). (**e**) Excess water and nutrient (Group E). (**f**) Water and nutrient deficit (Group F). (**g**) Moderate water and nutrient stress (Group G). (**h**) Without water and nutrient stress (Group H).

Morphological analysis is a visual inspection of plant health by an expert. We performed a morphological analysis to validate the effects of irrigation frequency on plants. Figure 5a shows a plant with excess water (Group A). The expert observed root suffocation causing defoliation (leaf fall), leaves with brown tips due to lack of iron, and compacted soil due to increased irrigation. Figure 5b shows a plant with a water deficit (Group B). The expert observed limitations of leaf development, withered leaves, dehydration, death of plant tissue (necrosis), and a decrease in chlorophyll. Figure 5c shows a plant without water stress (Group C). The expert observed an increment in green leaves, the development of new leaves without twisting, and firm stems. Figure 5d shows a plant with moderate stress (Group D). The expert observed green leaves and a lesser proportion of leaves in color brown due to the irrigation.

Figure 5e shows a plant with excess water and nutrient (Group E). The expert observed yellow leaves, burn edges, and blue–green leaves due to excess phosphorus and twisting of the leaves. Figure 5f shows a plant with water and nutrient deficits (Group F). The expert observed necrosis in the tissue of the leaves, twisting of the leaves, short lateral branches, and darker leaves. Figure 5g shows a plant with moderate water and nutrient stress (Group G). The expert observed firm stems and leaf growth. However, some leaves turned downward, with dark green and brown coloration, by phosphorus deficiency. Figure 5h shows a plant without water and nutrient stress (Group H). The expert observed the greatest leaf growth. Also, the leaves looked firm with green color by adequate photosynthetic activity. The stems remained firm and without leaf loss due to nitrogen and phosphorus. In our experiments, plants took up to 4 days to exhibit visual indicators, such as changes in color, texture, and physical alterations in the leaves.

### 4.2. Plant Dry Weight Analysis

**Figure 6:**
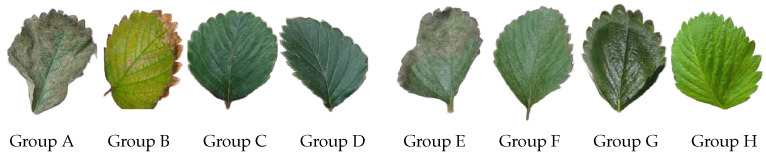
We see the condition of the leaves before beginning the drying process. Groups (A, B, E, F) with severe water stress will lose all their moisture in less time than groups (D, G) with moderate stress. On the other hand, in groups C and H, the leaves show a 100% healthy appearance due to humidity, so the drying time will be longer than that of the other groups.

We use a dry weight analysis to revalidate the stress detection in our strawberry plants dataset. This analysis is the weight recorded after drying plant tissue at temperatures higher than ambient temperature. This drying process eliminates water from the plant. The water is extracted from plant leaves until the water content is reduced to 0% using a surrounding air current. During the process, the sample sheet is weighed to obtain the drying curve that shows the Moisture Ratio (MR) over time [50,51]. Also, this analysis was used in previous works to detect stressed plants [52,53]. Figure 6 shows the leaves of our eight sets with plant stress phenotyping (A, B, C, D, E, F, G, H).

We use a Hamilton Beach 32100a dehydrator (Richmond, Virginia, U.S.) to temperatures of 40 °C, 50 °C, and 60 °C. The drying time at 40 °C was 4 h. In this case, some samples did not complete their dehydration. On the other hand, the drying time at 60 °C was 30 min, and some leaves showed burns due to the high temperature. The temperature that maintained the equilibrium of the dehydration curve of the leaves was 50 °C for 2 h. The samples were weighed every 15 min during the process. For that, we use a portable digital scale with a precision of 0.01 g. To apply the dry weight method, the leaves of stressed and unstressed plants have the same initial weight (300 mg). We take the weight every 15 min to calculate the MR and dehydration curve using Equation (11).

(11) $$MR=\frac{M_{t}-M_{e}}{M_{o}-M_{e}}$$

where $M_{t}$ denotes the humidity at time *t*, $M_{o}$ the initial humidity, and $M_{e}$ the equilibrium humidity. Table 3 shows the behavior of the dry weight in our eight sets of plants (A, B, C, D, E, F, G, H). Also, the results in bold refer to losing moisture slower. In the first 30 min, the leaves with excess water (Group A) and water deficit (Group B) similarly lost moisture. The leaves of group A lost all their humidity after 120 min, while the leaves of group B did so after 75 min. In this case, the drying time is longer in the leaves of plants with excess water. Group C leaves lost 10% of their moisture at 15 min, and at 75 min, they still retained 22%. The leaves of Group D with moderate stress had similar behavior to those of Group C, but they lost weight at a higher rate during the process.

On the other hand, Group E (excess of water and nutrients) lost moisture at a lower rate than Group F (deficit of water and nutrients). This trend was constant throughout the process. The leaves of Group G (moderate stress of water and nutrients) lost weight at a higher rate during the entire drying process compared to group H. Finally, group H behaved similarly to plants without stress in Group C. In this case, the same amount of moisture was retained for the majority of the process. However, by minute 75, Group C had lost less water. Although all the groups were dehydrated in 120 min, the dry weight analysis showed that the plants without stress retained the moisture for a longer time.

### 4.3. Dataset Discussion

**Figure 7:**
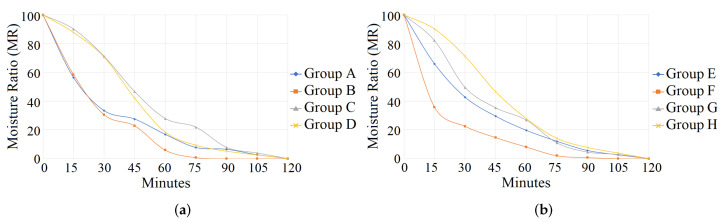
Image (**a**) shows that in the first 90 min, the leaves without stress in group C retained humidity in a higher proportion than group D. As expected, the leaves of groups A and B lost humidity faster due to stress, due to excess and deficit of water. Image (**b**) shows a similar behavior: in the first 60 min, leaves without stress in group H retain more humidity than those in group G, followed by groups E and F. (**a**) Plants with water irrigation. (**b**) Plants with water irrigation and nutrients.

In our morphological analysis, the expert observed an increment of green leaves in groups C and H, the development of new leaves, and firm stems. In groups A, B, D, E, F, and G, the plants presented leaves with brown tips, leaf fall, necrosis, withering, burn edges, etc. On the other hand, Figure 7 shows the behavior of the different groups with the dry weight analysis. In this analysis, the dry weight in the leaves of strawberries subjected to stress is significantly lower than without stress, i.e., groups C and H have a higher water retention performance in 0 to 90 min. These evaluations allow us to confirm that our images present plants with different stress phases. Furthermore, they corroborate the reliability of our training (Section 3.1) and our evaluation (Section 5). In these cases, the morphological and dry weight analyses were used to classify the stress of our sets. In our dataset, the unstressed plants had an average distance of 65 to 74 cm, and the stressed plants of 75 to 80 cm.

## 5. Discussion and Results

In this section, we present the experiments of our methodology for plant stress phenotyping using three-dimensional information and the hardware used. These experiments are the semantic segmentation evaluation (Section 5.2) and the 3D model evaluation (Section 5.3). For that, we use the “Eschikon Plant Stress Phenotyping Dataset” [43] and a proposed dataset of strawberry plants (Section 4).

### 5.1. Hardware

The hardware used in this study includes an Intel Core i9 CPU, an NVIDIA GeForce RTX 4070 GPU (Santa Clara, CA, USA), 64 GB of ADATA RAM, and an Intel Z790G ATX Gaming motherboard. This setup was chosen to ensure high computational performance and efficiency, particularly for handling complex deep learning models and data-intensive tasks. The Intel Core i9 CPU offers robust multi-core performance, while the NVIDIA RTX 4070 GPU provides the necessary graphical processing power to accelerate deep learning computations.

### 5.2. Semantic Segmentation Evaluation

We present the evaluation metrics, results, and discussions in our semantic segmentation step. For that, the quantitative evaluation was performed with recall, precision, and F-score measures. On the other hand, we use the “Eschikon Plant Stress Phenotyping Dataset” [43] and a proposed dataset of strawberry plants (Section 4).

#### 5.2.1. Semantic Segmentation Metrics

We use pixel comparison in our quantitative evaluation. This evaluation compares our semantic segmentation with the ground truth. To provide quantitative results, we used three measures (recall, precision, and F-score) based on the numbers of true positives (Tp), true negatives (Tn), false positives (Fp), and false negatives (Fn). The true positives Tp are the pixels whose labels are predicted correctly with respect to the ground truth of plant classification ($\upsilon^{1}$). The true negatives Tn are the correctly predicted labels with respect to the ground truth of the no-plant label ($\upsilon^{2}$). The false positives Fp correspond to all those pixels whose label is incorrect. Finally, false negatives Fn correspond to those pixels where the prediction did not assign the corresponding plant classification ($\upsilon^{1}$). On the other hand, a five-fold cross-validation was applied, dividing the dataset into five parts and alternating the validation subset in each iteration. Tables 6 and 7 show the cross-validation results. This cross-validation approach allows for a robust evaluation and minimizes overfitting in the results, enhancing the model’s generalization.

We use three measures in our semantic segmentation evaluation (recall, precision, and F-score) Equations (12)–(14). In this sense, we used recall to measure the proportion of pixels whose semantic label was predicted correctly regarding the total amount of pixels in its ground truth label—that is, in simple terms, the amount of ground truth that was predicted correctly. On the other hand, precision measures the proportion of semantic labels that were predicted correctly. We employed specificity (SP) to measure the proportion of true negatives correctly identified in all negative cases—that is, the probability that the test is classified as negative when it is negative. The jaccard index (JI) is utilized to evaluate the similarity and diversity of the predicted labels compared to the ground truth labels. The jaccard index is calculated as the number of values belonging to both sets (intersection) divided by the unique number across both sets (union). Accuracy (AC) is the proportion of correct predictions (Tp and Tn) divided by the number of examined cases. Finally, F-score helps to summarize the performance of the predictions returned by the system. In sum, we could say that for a system with good performance, both the recall and precision should tend to be one, meaning that most of the system’s predictions tend to be correct and that such predictions tend to cover most of the ground truth. If this is the case, then F-score should tend to be one.

(12) $$recall=\frac{Tp}{Tp+Fn}$$

(13) $$precision=\frac{Tp}{Tp+Fp}$$

(14) $$F-score=2\frac{recall\times precision}{recall+precision}=\frac{Tp}{Tp+\frac{1}{2}\left(Fp+Fn\right)}$$

(15) $$accuracy=\frac{Tp+Tn}{Tp+Tn+Fp+Fn}$$

(16) $$jaccard\;index=\frac{Tp}{Tp+Fp+Fn}$$

The Area Under the Curve (AUC) provides a measure of the model to discriminate between the classes. This metric represents the area under the receiver operating characteristic (ROC) curve, which plots the true positive rate against the false positive rate at various threshold settings, where f(x) is a receiver operating characteristic curve in which the true-positive rate (SE) is plotted in the function of the false-positive rate (1-SP) for different cut-off points. The Polygon Area Metric (PAM) [54] is calculated by determining the area of the polygon formed by the points representing RE/SE, SP, JI, AC, F1, and AUC within a regular hexagon. It is important to note that the regular hexagon consists of 6 sides, each with a length of 1, and the total area of the hexagon is 2.59807. The lengths from the center towards the hexagon vertex correspond to the values of RE/SE, SP, JI, AC, F1, and AUC, respectively, where PA represents the area of the formed polygon. It is important to mention that to normalize the PAM within the [0, 1] range, the PA value is divided by 2.59807.

(17) $$\mathrm{AUC}=\int_{0}^{1}f\left(x\right)\;dx$$

(18) $$\mathrm{PAM}=\frac{PA}{2.59807}$$

#### 5.2.2. Semantic Segmentation Results

We use two different datasets to evaluate semantic segmentation, the “Eschikon Plant Stress Phenotyping” dataset [43] and our proposed dataset (Section 4). The proposed dataset was obtained from strawberry plants and includes 1440 images with a resolution of 640 × 480 pixels. Table 4 shows the result of training the network with different amounts of images. Experiments with 75,000 images showed the highest segmentation and detection precision in the leaves of the plant.

Our methodology in the semantic segmentation step obtained the best performance with 75,000 images of training (Table 4). For example, our plant segmentation had an average recall of 0.95, i.e., considering the ground-truth, we recognized 95%. The plant segmentation had an average precision of 0.935, i.e., in the semantic segmentation, we segmented 93.5% correctly. Also, this semantic segmentation had an average F-score of 0.94. The segmentation is fundamental since a correct recognition provides a suitable 3D extraction because the recognition is proportional to the precision of 3D reconstruction on the (X, Y) axis of the 3D model. Table 4 shows quantitative results with “Eschikon Plant Stress Phenotyping Dataset” [43] and a proposed dataset. The results in bold refer to the best score. Figure 3b shows a qualitative result of our methodology in semantic segmentation. We can see that this qualitative result presents a proper classification of the plant.

On the other hand, although there exist semantic segmentation methods that consider green areas, they are predominantly trained on non-stressed conditions—that is, although they can detect plants, they have limitations in recognizing crops under stress. Furthermore, the majority of these networks focus on spatial information from urban outdoor environments, which can impact the accuracy of detection when using a top-down view of plants, as in our proposed methodology. Motivated by these limitations, we have developed a new configuration for plant segmentation with and without stress. Also, our semantic segmentation architecture allows us to convert a dataset with few images into a big training set. This configuration provides flexibility to our proposal, as we could easily consider other environments or crops.

### 5.3. Three-Dimensional Model Evaluation

We present the evaluation metrics, results, and discussions in our 3D reconstruction step. For that, the quantitative evaluation was performed with the Root Mean Squared (RMS) error. On the other hand, we use the proposed dataset of strawberry plants (Section 4). Finally, we discuss our results using the evaluation metrics.

#### 5.3.1. Three-Dimensional Model Metrics

We use a Kinect sensor to obtain our strawberry plant stress phenotyping dataset (Section 4). In our dataset, each RGB image has a depth image. This depth has values ranging from 0 to 255 (gray-scale value). We use a top perspective where the gray-scale tends to 0 when the distance increases between the camera and the plant. In our experiments, the unstressed plants were an average distance of 65 to 74 cm and the stressed plants of 75 to 80 cm. We present the evaluation of the strawberry plant stress ranges in Section 4.

The mean square error (RMS) determines how much the actual data differ from the predictions made by a model. In the 3D reconstruction step, we compared the centroids of predictions made by our model with the ground truth reconstruction. For that, we used the RMS error Equation (19); where $d_{k}$ and $d_{k}^{gt}$ are the centroids (X,Y,Z) predicted by our methodology and its ground-truth, respectively. *T* is the total number of images in all the dataset, and *k* is the images index.

(19) $$RMS=\sqrt{\frac{1}{T}\sum_{k}\;{\left(d_{k}^{gt}-d_{k}\right)}^{2}}$$

#### 5.3.2. Three-Dimensional Model Results

We use the proposed dataset of strawberry plants (Section 4). Also, we compared the centroids (X,Y,Z) of each leaf in the 3D model of the ground truth with our centroids (X,Y,Z). The mean square error (RMS) determines how much the actual data differ from the predictions made by a model. Table 5 shows the RMS obtained by comparing the centroids (X,Y,Z) of the ground truth with respect to the centroids (X,Y,Z) of the CNN. In this experiment, we have an average RMS error (Z) of 0.069241, i.e., an error of 0.069 cm in 4 m. On the other hand, considering the coordinates (X,Y,Z) in the extraction, we had an average RMS (X,Y,Z) of 0.029124. Table 5 shows our 3D model evaluation. On the other hand, we outperform the average performance by comparing the proposed approach with a 3D reconstruction approach that uses a depth sensor (PSR [2]).

**Figure A1:**
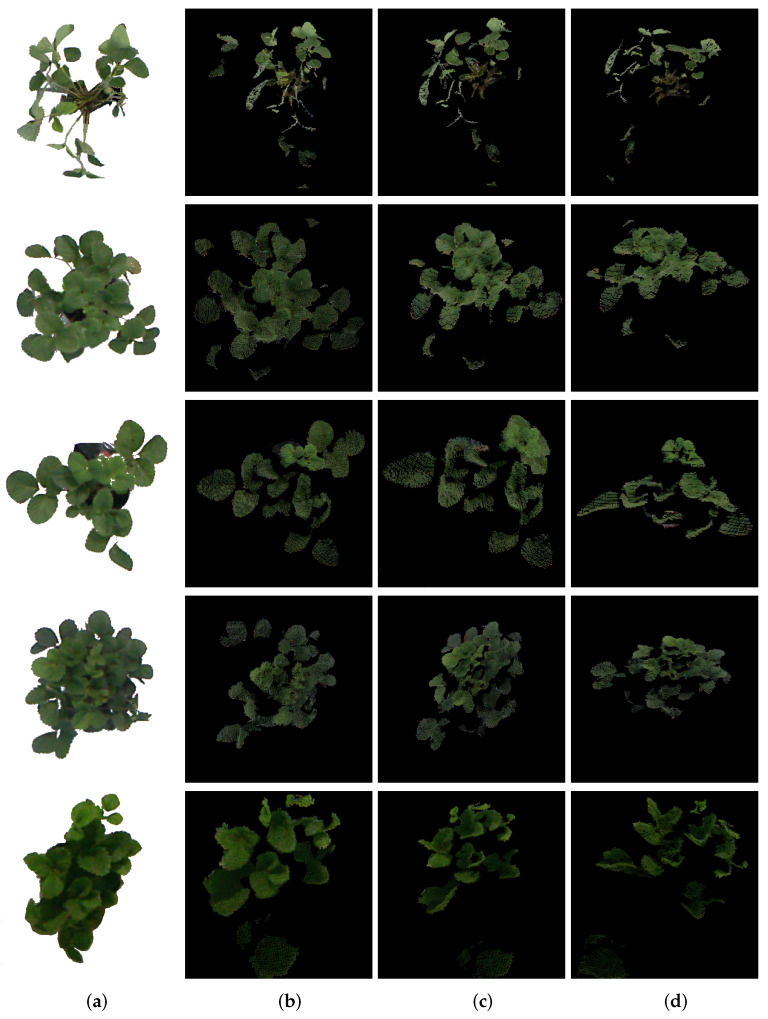
Strawberries images (**a**) and 3D Models (**b**). Qualitative results of our 3D reconstruction from diverse perspectives. (**a**) Input image. (**b**) 3D reconstruction. (**c**) 3D reconstruction. (**d**) 3D reconstruction.

Unlike previous works, the proposed methodology only requires an RGB image to perform stress analysis, i.e., we do not need depth sensors to obtain the three-dimensional reconstruction. This allows us to provide a more accessible tool to rural producers. Additionally, the network used for depth inference has low computational resource consumption, eliminating the need for high-performance equipment. Figure A1 shows a qualitative result of our methodology in 3D reconstruction. We can see that this qualitative result presents a proper reconstruction of the plant.

#### 5.3.3. Three-Dimensional Model vs. 2D Classification

We compare 3D and 2D classification approaches for plant stress detection. For that, we train four approaches with the same number of images. In the 2D approaches, we use the YOLOv4 [55] and YOLOv8 [56] networks. In addition, we compare our results with a 3D approach (PSR [2]) for detecting stressed plants. Table 6 shows the quantitative results of the four different methods. Also, the results in bold refer to the best score.

Although the 2D and 3D approaches internally process different information for plant stress inference, the proposed 3D method and the 2D approaches use the same input information. Also, these works provide the same output binary classification. This binary classification allows us to make quantitative comparisons between the results.

We use the YOLOv4 [55] and YOLOv8 [56] networks as the 2D approaches. YOLO has high performance in detection accuracy and processing time. However, this approach requires large datasets for training. Unlike our methodology, we can convert a small set of images to a large training set (Section 3.1.2). This increase in data allows us to outperform the 2D approaches. In Table 6, the proposed methodology has a difference of 0.1873 higher recall, 0.1738 higher precision, 0.1804 higher F1-score, and 0.1796 higher accuracy than the 2D classification method. Although the 2D network increases its classification as the number of images increases, its performance is lower than our proposal that considers three-dimensional information. The latter is because the 2D method can recognize stress in images where the plant shows obvious physical signs of stress. However, in a plant with the first days of stress, the physical signs are not visible visually, while the decline of the leaves is one of the first morphological symptoms of crop stress. This allows our 3D methodology to detect stress even when visual signals have not appeared on the plant.

We compare the proposed methodology with a three-dimensional model for detecting stressed plants [2]. This model utilizes a depth sensor for analyzing plants under stress. The use of RGBD sensors simplifies the process of three-dimensional reconstruction. However, while depth sensors simplify 3D extraction, they have limitations. Their applicability is primarily confined to indoor environments, and they can increase implementation costs. For instance, these sensors often fail in outdoor situations due to solar radiation. Also, they are not integrated into personal devices (such as mobile phones, personal assistants, or personal computers). Lastly, their power consumption (in watts), cost, and size are higher than those of RGB sensors. In Table 6, the proposed methodology has a difference of 0.0249 higher recall, 0.0068 lower precision, 0.0088 higher F1-score, and 0.0091 higher Accuracy than the 3D classification method with a depth sensor.

Table 7 presents additional classification metrics, including specificity, jaccard index, AUC, and PAM. These metrics are essential for calculating the PAM score, which provides a general metric of the performance of each approach compared to basic precision, recall, and F1-score metrics. The proposed approach demonstrates significant improvements across these metrics. In the case of 800 images, our 3D model achieved the most elevated specificity (0.9432), jaccard index (0.8763), and AUC (0.9414), surpassing other models. For the case of 1000 images, our 3D model showed the highest values in most of the metrics, jaccard index (0.8258), accuracy (0.9084), and AUC (0.9098). Additionally, our 3D model reached a PAM of 0.8684 and 0.9017 for 800 and 1000 images, respectively, surpassing the general performance of other models.

**Figure 8:**
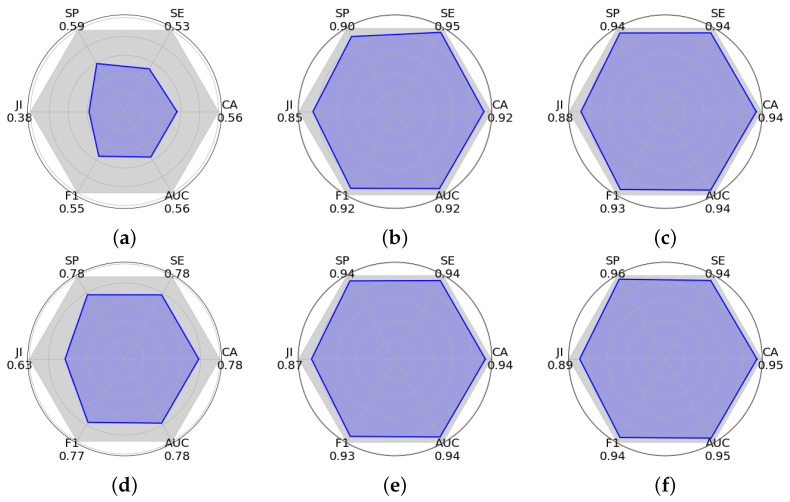
Comparative evaluation of various neural network architectures using Polygon Area Metric (PAM). The metrics were calculated using the average confusion matrices from Figure A2. The plots show six key performance indicators—Specificity (SP), Sensitivity (SE), Jaccard Index (JI), Accuracy (CA), Area Under the Curve (AUC), and F1-score—with PAM representing the blue shaded area inside each polygon. The larger polygon area indicates better overall performance according to PAM. (**a**) YOLOv8 (800). (**b**) PSR (800). (**c**) Our 3D model (800). (**d**) YOLOv8 (1000). (**e**) PSR (1000). (**f**) Our 3D model (1000).

Figure 8 shows different evaluations for classifying crops using the graphic PAM. This metric represents an overall measure of performance, calculated from six key indicators: specificity, sensitivity, jaccard index, accuracy, AUC, and F1-score. Each plot shows a polygon where the blue-shaded area represents the PAM score. A larger shaded area indicates a better general performance. In these experiments, our 3D model exhibits the largest shaded area, achieving PAM scores of 0.8684 for 800 images and 0.9017 for 1000 images. Other models (PSR) show acceptable performance, although lower. The architecture YOLOv8 exhibits smaller areas, reflecting lower performance. These results underline the effectiveness of our 3D model in classification tasks, demonstrating balanced and superior performance compared to other architectures.

Table 8 shows the processing time of the different classification approaches. The fastest approaches were YOLOv4 [55] and YOLOv8 [56]. However, in our experiments, these approaches present differences of 0.2094 and 0.1804 lower performance in F1-score, respectively, than our proposed methodology (see Table 6). The PSR [2] approach has processing times similar but weaker than our approach since it simplifies 3D reconstruction using depth sensors. However, their performance is lower than that of our approach (see Table 6).

**Figure 9:**
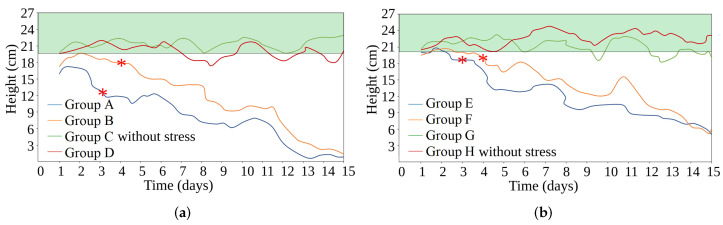
The images show the height variation in the foliage of our strawberry plants during the 15-day evaluation period. The maximum foliage height is 27 cm. The green area represents the without-stress threshold, and the red asterisks indicate the day when the plants exhibit visual signs of stress. Also, our method detected an average height of 20 cm in the foliage of plants from groups C and H. (**a**) Plants with water irrigation. (**b**) Plants with water irrigation and nutrients.

On the other hand, although there are two-dimensional (2D) analysis techniques that can detect plant stress, these techniques rely on visual information of stress signs. On the other hand, our methodology detects a variation in leaf height from the first day, indicating signs of stress in the plant, and this variation increases as the day progresses (see Figure 9). In contrast, plants took up to 4 days to exhibit visual indicators, such as changes in color, texture, and physical alterations in the leaves. In other words, a 2D technique would require these additional days to begin detecting stress in the leaves. This increase in response time highlights an advantage in our methodology analyzing information in three dimensions. Figure 9 shows the variation in leaf heights among different groups during the 15-day evaluation period. The green area represents the without-stress threshold, and the red asterisks indicate the day when the plants exhibit visual signs of stress.

## 6. Conclusions

In this work, we introduced a new approach for plant stress using deep learning and 3D reconstruction from a single RGB image. Our strategy was to divide and simplify the 3D plant extraction process. This strategy combines the abstraction power of deep learning and the information that provides crop geometry. For that, our methodology has three steps. First, the plant recognition step provides the segmentation, location, and delimitation of the crop. Second, we proposed a leaf detection analysis to classify and locate the boundaries between the different leaves. Third, we used a Deep Neural Network (DNN) and the 3D reconstruction for plant stress detection.

The quantitative experiments comprised the plant recognition (semantic segmentation) and its 3D extraction. In the recognition evaluation, we used two datasets that provide different crops (“Eschikon Plant Stress Phenotyping” dataset and a proposed dataset). For that, we analyzed two labels of crops (plant and no-plant). For example, our plant segmentation had an average recall of 0.95, i.e., considering the ground-truth, we recognized 95%. On the other hand, the plant segmentation had an average precision of 0.935, i.e., considering the semantic segmentation, we segmented 93.5% correctly. The segmentation is fundamental since the plant recognition is proportional to the precision of 3D extraction on the (X,Y) axis of the 3D model.

In the 3D plant extraction evaluation, we used our proposed dataset. We used the RMS error for the quantitative evaluation. The Mean Square Error (RMS) determines how much the actual data differ from the predictions made by a model. For that, we compared the centroids (X,Y,Z) of each leaf in the 3D model of the ground truth with our centroids (X,Y,Z). In this experiment, we had an average RMS error (Z) of 0.069241, i.e., an error of 0.069 cm in 4 m. On the other hand, considering the coordinates (X,Y,Z) in the extraction, we had an average RMS (X,Y,Z) of 0.029124.

We compared our 3D methodology with a 2D classification method using deep learning. Our plant stress detection had an average recall of 0.9661, i.e., considering the ground-truth, we detected 96.61%, while the previous work was 77.88%. Also, we had an average precision of 0.9344, i.e., considering our stress detection, we detected 93.44% correctly, while the previous work was 76.06%. In other words, the proposed methodology had 24.05% higher recall and 22.86% higher precision than the 2D classification method. The latter is because the 2D method can recognize stress in images where the plant shows obvious physical signs of stress. However, in a plant with the first day of stress, the physical signs are not visible visually. The decline of the leaves is one of the first morphological symptoms of crop stress. This allowed our 3D methodology to detect stress even when visual signals had not appeared on the plant.

Finally, we concluded that our methodology allows plant stress detection using visual characteristics and a single RGB image. By employing an RGB camera for crop analysis, we can deploy the methodology across multiple devices, improving portability and reducing implementation costs. These characteristics enable remote implementation of the methodology, which can facilitate its adoption in rural areas. On the other hand, we studied the case of strawberry crops in an image where the camera looks at a top view. For that, we worked with different databases such as “Eschikon Plant Stress Phenotyping” and a proposed dataset. To our knowledge, the proposed approach is the first work that has detected stress in crops using 3D information from a single RGB image.

## Figures and Tables

**Table 1:** Model performance as a function of the number of superpixels.

	Number of Superpixels				
	800	900	999	1100	1200
Precision	0.9056	0.9235	0.9344	0.9392	0.9286
Recall	0.9314	0.9441	0.9661	0.9551	0.9548
F1-Score	0.9183	0.9337	0.9500	0.9471	0.9415
Accuracy	0.9341	0.9455	0.9591	0.9568	0.9523

**Table 2:** We propose an irrigation scheme with different time intervals to generate stress and non-stress conditions in strawberry plants. The groups (A, B, C, D) were irrigated with water, while the groups (E, F, G, H) were irrigated with water and nutrients.

	Group	Dose	Watering (Days)	Category
water	A	250 mL	1	excess
	B	250 mL	5	deficit
	C	250 mL	2	without stress
	D	250 mL	3	moderate stress
water and nutrient	E	250 mL NPK	1	excess
	F	250 mL NPK	5	deficit
	G	250 mL NPK	2	moderate stress
	H	250 mL NPK	3	without stress

**Table 3:** This table shows the behavior of the dry weight in our eight sets of plants (A, B, C, D, E, F, G, H). The dry weight method confirmed that the non-stressed leaves (C, H) and the moderately stressed leaves (D, G) retain a higher percentage of moisture as the drying time progresses. The leaves of stressed plants (A, B, E, F) show the opposite behavior, losing moisture faster than healthy leaves.

Time	Water				Water and Nutrient			
	A	B	C	D	E	F	G	H
0	100	100	100	100	100	100	100	100
15	56	58	90	88	66	36	82	90
30	33	31	71	71	43	22	50	71
45	27	23	46	42	29	15	35	46
60	17	6	28	18	20	8	27	28
75	8	1	22	9	12	2	11	14
90	6	0	8	5	6	1	5	8
105	3	0	4	3	3	0	3	4
120	0	0	0	0	0	0	0	0

**Table 4:** Semantic segmentation of plant. We use three measures (Recall, Precision, and F-score). Also, we use the “Eschikon Plant Stress Phenotyping Dataset” [43] and a proposed dataset of strawberry plants (Section 4).

Images	Dataset Sugarbeet [43]			Dataset Strawberry		
	Precision	Recall	F1-Score	Precision	Recall	F1-Score
20,000	0.78	0.80	0.78	0.85	0.91	0.87
25,000	0.83	0.85	0.83	0.90	0.93	0.91
30,000	0.85	0.90	0.87	0.91	0.93	0.91
40,000	0.88	0.93	0.90	0.91	0.94	0.92
50,000	0.91	**0.94**	0.92	0.93	0.95	0.93
**75,000**	**0.93**	**0.94**	**0.93**	**0.94**	**0.96**	**0.95**

**Table 5:** Three-dimensional model. We compare the centroids of the coordinates (X,Y,Z) of 3D models with the ground-truth. We use the RMS error to measure the 3D model results. Also, we measure the RMS error in meters.

	RMS (*X*)	RMS (*Y*)	RMS (*Z*)	Average
PSR [2]	0.013416	0.029814	**0.007843**	0.03697
Our 3D model	**0.009217**	**0.008913**	0.069241	**0.02912**

**Table 6:** Three-dimensional model vs. 2D classification. We use four measures (Recall, Precision, F-score, and Accuracy). Also, we use our proposed dataset of strawberry plants (Section 4).

	800 Images				1000 Images			
	Pre.	Rec.	F1-Sc.	Acc.	Pre.	Rec.	F1-Sc.	Acc.
YOLOv4 [55]	0.5574	0.4985	0.5263	0.5359	0.7302	0.7512	0.7406	0.7500
YOLOv8 [56]	0.5733	0.5260	0.5486	0.5578	0.7606	0.7788	0.7696	0.7795
PSR [2]	0.8910	**0.9521**	0.9205	0.9249	**0.9412**	0.9412	0.9412	0.9500
Our 3D model	**0.9239**	0.9444	**0.9341**	**0.9438**	0.9344	**0.9661**	**0.9500**	**0.9591**

Numbers in bold indicate the best scores, where higher is better.

**Table 7:** Three-dimensional model vs. 2D classification. We use four measures (Specificity, Jaccard Index, AUC, and PAM). Also, we use the our proposed dataset of strawberry plants (Section 4).

	800 Images				1000 Images			
	Spe.	J. I.	AUC	PAM	Spe.	J. I.	AUC	PAM
YOLOv4 [55]	0.5761	0.3571	0.5374	0.2545	0.7489	0.5880	0.7496	0.5219
YOLOv8 [56]	0.5911	0.3780	0.5587	0.2769	0.7802	0.6255	0.7790	0.5674
PSR [2]	0.9020	0.8528	0.9241	0.8343	**0.9565**	0.8889	0.9488	0.8816
Our 3D model	**0.9432**	**0.8763**	**0.9414**	**0.8684**	0.9544	**0.9048**	**0.9555**	**0.9017**

Numbers in bold indicate the best scores, where higher is better.

**Table 8:** Average processing times (seconds) for different stress detection approaches. This comparison uses our proposed dataset of strawberry plants (Section 4).

Approach	Proposed Dataset
YOLOv4 [55]	0.108
YOLOv8 [56]	**0.071**
PSR [2]	4.7830
Our 3D model	6.456

Numbers in bold indicate the best scores, where lower is better.

**Figure A2:**
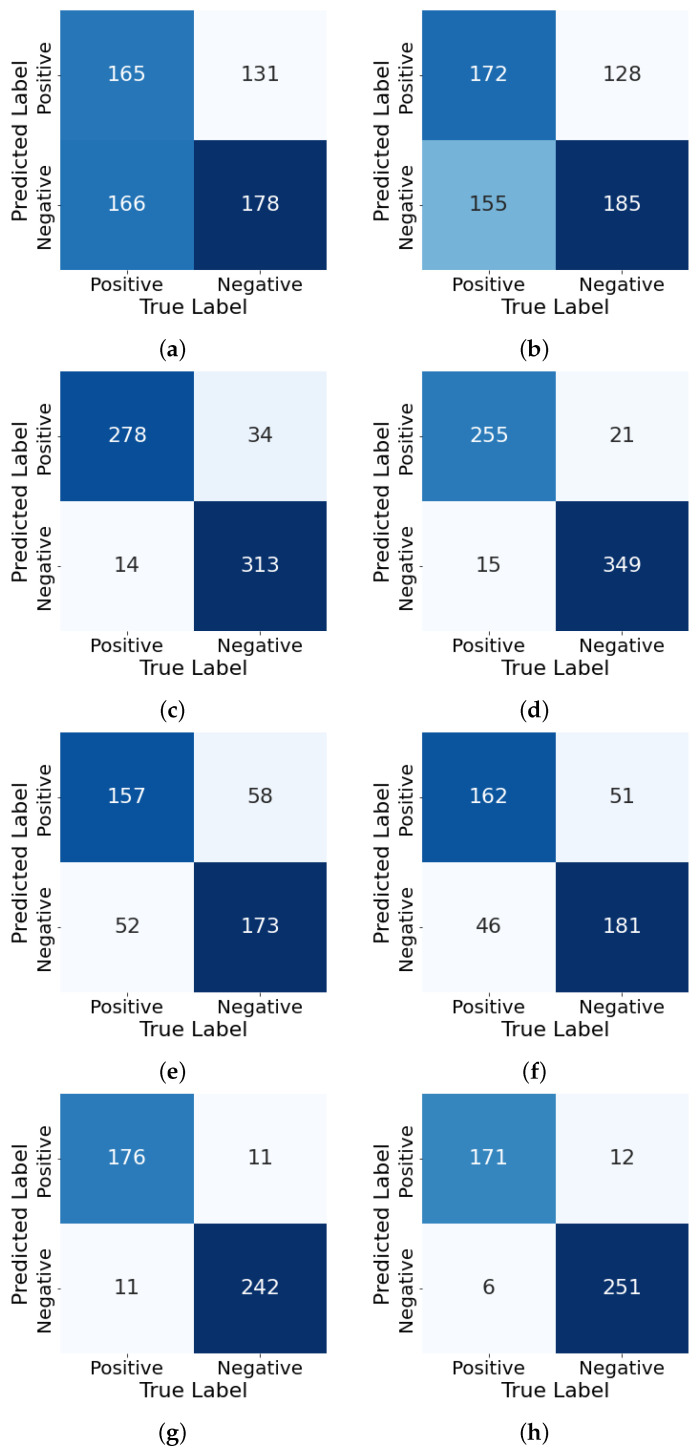
Average confusion matrices for crop classification from cross-validation (5-fold) using different models for plant stress detection. (**a**) YOLOv4 (800). (**b**) YOLOv8 (800). (**c**) PSR (800). (**d**) Our 3D model (800). (**e**) YOLOv4 (1000). (**f**) YOLOv8 (1000). (**g**) PSR (1000). (**h**) Our 3D model (1000).

**Figure A3:**
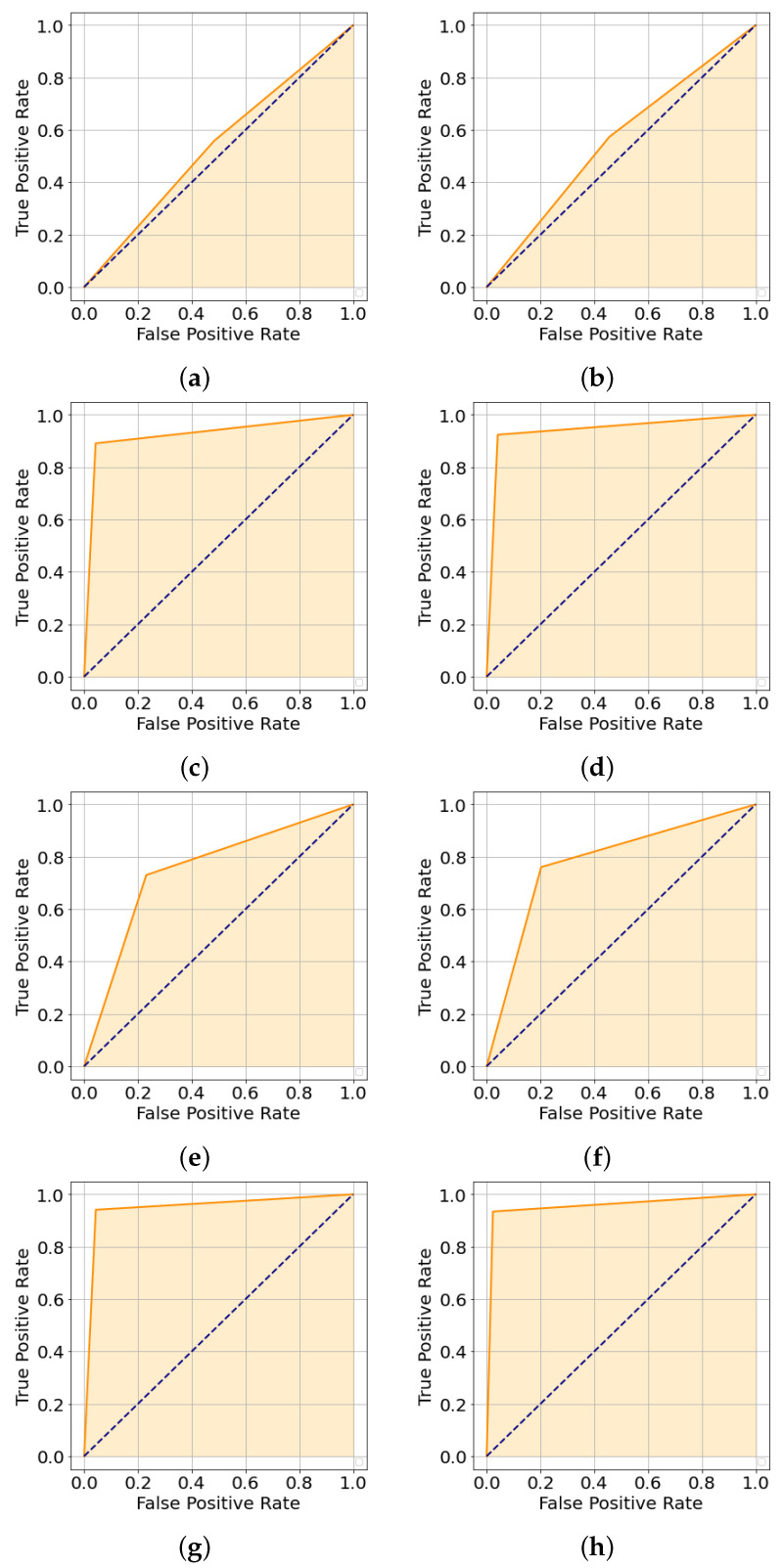
ROC curves and corresponding AUC values for fern classification using different models for plant stress detection. The metrics were calculated using the average confusion matrices from Figure A2. Each plot shows the True Positive Rate (TPR) against the False Positive Rate (FPR), with the diagonal line representing a random guess. The area under each curve (AUC) reflects the ability of the models to distinguish between classes. (**a**) YOLOv4 (800). (**b**) YOLOv8 (800). (**c**) PSR (800). (**d**) Our 3D model (800). (**e**) YOLOv4 (1000). (**f**) YOLOv8 (1000). (**g**) PSR (1000). (**h**) Our 3D model (1000).

## Data Availability

The datasets generated and analyzed during the current study are not publicly available.
